# Stepwise introduction of stabilizing mutations reveals nonlinear additive effects in *de novo* TIM barrels

**DOI:** 10.1002/pro.4926

**Published:** 2024-02-21

**Authors:** Johanna‐Sophie Koch, Sergio Romero‐Romero, Birte Höcker

**Affiliations:** ^1^ Department of Biochemistry University of Bayreuth Bayreuth Germany

**Keywords:** (β/α)_8_‐barrel, additive effects, asymmetry, de novo protein design, DeNovoTIMs, protein folding, TIM barrel

## Abstract

Over the past decades, the TIM‐barrel fold has served as a model system for the exploration of how changes in protein sequences affect their structural, stability, and functional characteristics, and moreover, how this information can be leveraged to design proteins from the ground up. After numerous attempts to design *de novo* proteins with this specific fold, sTIM11 was the first validated *de novo* design of an idealized four‐fold symmetric TIM barrel. Subsequent efforts to enhance the stability of this initial design resulted in the development of DeNovoTIMs, a family of *de novo* TIM barrels with various stabilizing mutations. In this study, we present an investigation into the biophysical and thermodynamic effects upon introducing a varying number of stabilizing mutations per quarter along the sequence of a four‐fold symmetric TIM barrel. We compared the base design DeNovoTIM0 without any stabilizing mutations with variants containing mutations in one, two, three, and all four quarters—designated TIM1q, TIM2q, TIM3q, and DeNovoTIM6, respectively. This analysis revealed a stepwise and nonlinear change in the thermodynamic properties that correlated with the number of mutated quarters, suggesting positive nonadditive effects. To shed light on the significance of the location of stabilized quarters, we engineered two variants of TIM2q which contain the same number of mutations but positioned in different quarter locations. Characterization of these TIM2q variants revealed that the mutations exhibit varying effects on the overall protein stability, contingent upon the specific region in which they are introduced. These findings emphasize that the amount and location of stabilized interfaces among the four quarters play a crucial role in shaping the conformational stability of these four‐fold symmetric TIM barrels. Analysis of *de novo* proteins, as described in this study, enhances our understanding of how sequence variations can finely modulate stability in both naturally occurring and computationally designed proteins.

## 1 INTRODUCTION

Proteins are exceptional macromolecules that exhibit a high diversity of structural topologies, enabling them to execute a multitude of central biological functions and regulate essential processes within living organisms. To perform their functions, proteins have to fold into native conformations and be stable (Anfinsen, 1973; Braselmann et al., 2013; Englander and Mayne, 2014; Munson et al., 1996). Protein stability is a fundamental biological parameter that results from a delicate balance of forces between the protein and its environment (solvent, ions, macromolecules, etc.), and plays an important role in various biochemical aspects such as expression, solubility, evolution, and functionality (Bloom et al., 2006; Goldenzweig and Fleishman, 2018; Nisthal et al., 2019). A fine balance of stability, dynamics, and function shapes the protein sequence space accessible for a specific fold, both for natural and designed proteins (Koehl and Levitt, 2002; Linsky et al., 2022). Therefore, analyses of the effects of sequence on protein folding and stability are crucial to increase our understanding of the molecular determinants of sequence–structure–stability relationships.

These fundamental aspects have been explored by comprehensive analyses of different protein folds. The TIM‐barrel fold, also known as the (β/α)_8_‐barrel, stands as a prominent model system in these studies. Its topology is a highly versatile and an abundant architecture in nature as it is adopted by around one‐tenth of known proteins and can be found in six out of seven enzyme classes (Nagano et al., 2002; Romero‐Romero et al., 2021b; Sterner and Höcker, 2005). Triosephosphate isomerase, a central enzyme in glycolysis, was the first protein for which the (β/α)_8_‐barrel topology was described and hence became eponymous for this fold (Banner et al., 1975). Typically, TIM‐barrel proteins comprise 200–250 residues which assemble into eight repeating (βα) units. The elements within one unit are connected by a βα‐loop, while the individual units are interconnected by αβ‐loops. The barrel structure is then formed by assembly of the β‐strands into a circular parallel β‐sheet surrounded by α‐helices (Wierenga, 2001). It has been found that residues involved in catalysis cluster in regions that include the C‐terminal ends of the β‐strands and the βα‐loops (Sterner and Höcker, 2005). Meanwhile, residues responsible for preserving the fold stability, cluster in the core and in the αβ‐loops (Luger et al., 1990; Wiederstein and Sippl, 2005). The conformational and folding properties of this fold as well as its evolution have been thoroughly analyzed, revealing valuable insights into various aspects of the sequence–stability–function interplay in proteins (Chan et al., 2017; Chan et al., 2020; Halloran et al., 2019; Höcker et al., 2001; Höcker et al., 2004; Jain et al., 2021; Matthews and Crisanti, 1981; Miles et al., 1982; Richard, 2019; Romero‐Romero et al., 2015; Romero‐Romero et al., 2018; Sánchez del Pino and Fersht, 1997).

Likewise, this protein fold has been a suitable model system for *de novo* design and engineering approaches (Huang et al., 2016; Romero‐Romero et al., 2021b). Regarding the design of TIM barrels from scratch, sTIM11 was reported as the first structurally validated *de novo* TIM barrel, presenting a four‐fold symmetry and a sequence significantly different from the ones found in naturally occurring TIM barrels (Huang et al., 2016). This scaffold has been diversified to generate a collection of proteins focused on increased stability by a hydrophobic repacking (DeNovoTIMs) (Romero‐Romero et al., 2021a), the introduction of salt‐bridge clusters (DeNovoTIMs‐SB) (Kordes et al., 2022), or the addition of secondary‐structure elements on top of the barrel (Kordes et al., 2023; Wiese et al., 2021). In the DeNovoTIM family, significant nonadditive effects in thermal and conformational stability were observed when stabilizing mutations from different regions of the barrel were combined. However, it is uncertain whether the effects of the stabilizing mutations are additive when they are located in the same region of the barrel but introduced quarter by quarter in the sequence. Therefore, in this work, we investigate the contributions of stabilizing mutations by quarter‐wise addition from the previously reported DeNovoTIM0 (with no stabilizing mutations) to DeNovoTIM6 (stabilized in all four quarters) constructs. These newly engineered TIM quarter variants provide insights into the cumulative impact on stability properties and the effects of introducing asymmetry within this fold.

## 2 RESULTS AND DISCUSSION

### 2.1 Building *de novo* TIM barrels by quarter‐wise incorporation of stabilizing mutations

The base design in this work, DeNovoTIM0, is a previously characterized successor of the first validated *de novo* TIM barrel sTIM11, an idealized design with four‐fold symmetry (Huang et al., 2016). DeNovoTIM0 differs from sTIM11 with the mutations W34V and A38G (in all symmetry‐related quarters) and the additional point mutations C8Q and C181V. The modular design approach described in previous work followed a hydrophobic repacking strategy that led to a collection of DeNovoTIMs, whose variants contain stabilizing mutations in different regions of the barrel (single‐region designs) as well as combinations of the experimentally most stable variants (double‐ and triple‐region designs) (Romero‐Romero et al., 2021a). From the single‐region variants, the thermodynamic parameters from DeNovoTIM0 (containing no stabilizing mutations) to DeNovoTIM6 (stabilized in all four quarters) showed significant improvements both in thermal and conformational stability as well as other biophysical properties (>45°C shift in *T*_m_ and a ΔΔ*G* > 6 kcal mol^−1^).

**FIGURE 1:**
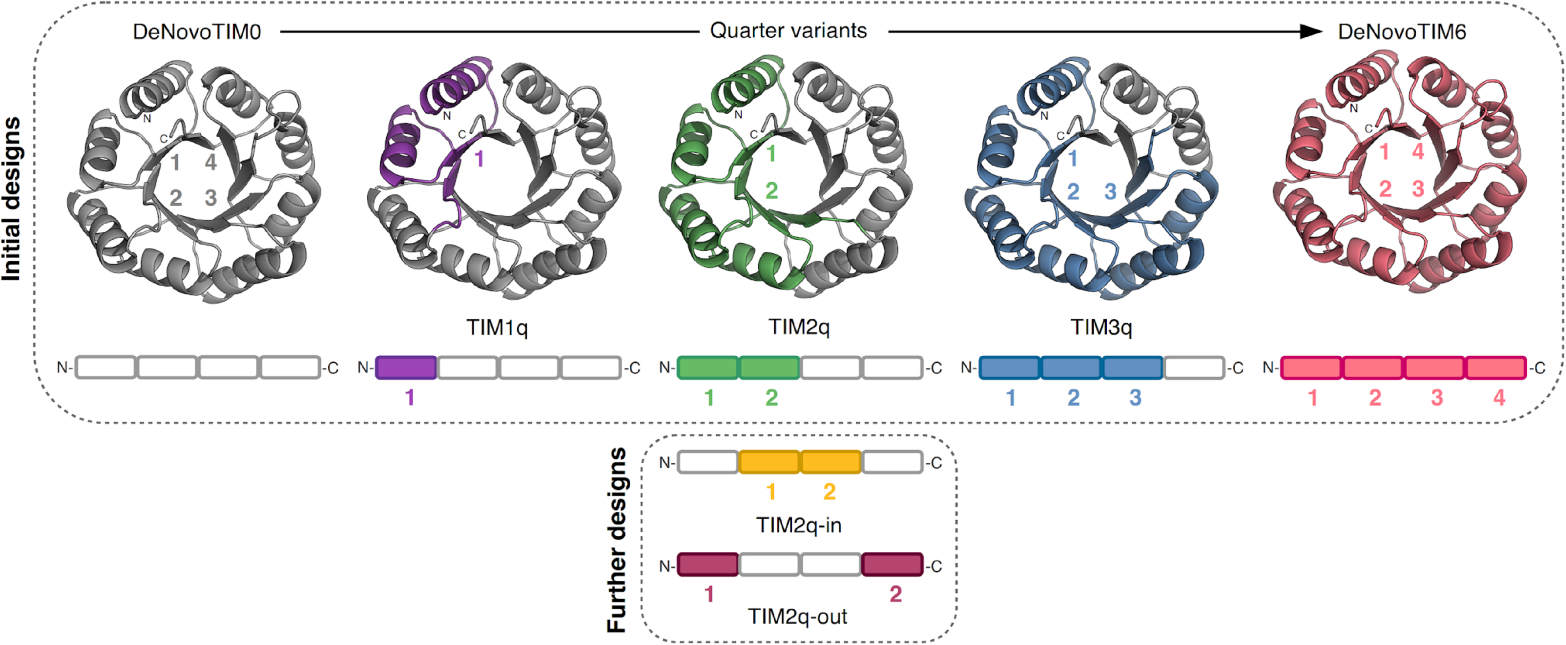
Design strategy of quarter variants from DeNovoTIM0 to DeNovoTIM6. The *de novo* TIM variants follow a quarter‐wise insertion of mutations Q11I, E15L, T18K, K31Q, and V34L which had a great stabilizing effect for DeNovoTIM6 compared to DeNovoTIM0. Each protein is represented as a structural model in which the N‐ and C‐termini as well as the number of quarters are indicated. Further, the variants are shown as a simplified peptide sequence in which one rectangle represents one quarter. For both schematics, quarters containing stabilizing mutations are highlighted in color and with the corresponding number. From left to right are shown: DeNovoTIM0 (gray) as the base design, TIM1q (purple) with one mutated quarter, TIM2q (green) with the first and second quarter mutated, TIM3q (blue) in which quarters one to three are stabilized, and DeNovoTIM6 (pink) which is fully stabilized. Further designs are variants of TIM2q and focus on variations in the location of the two quarters carrying the mutations. According to the location within the sequence, the proteins are named TIM2q‐in (yellow) and TIM2q‐out (red). See Table S1 for sequence information.

In comparison to DeNovoTIM0, DeNovoTIM6 contains five stabilizing mutations per quarter (Q11I, E15L, T18K, K31Q, and V34L, and equivalent positions in other quarters). Thus, in order to assess the additive effects of these mutations along the sequence, we introduced the mutations progressively from the N‐terminus to the C‐terminus quarter by quarter. The addition of the stabilizing mutations Q11I, E15L, T18K, K31Q, and V34L in the first quarter of DeNovoTIM0 generated the variant TIM1q (the number indicates the number of quarters carrying the mutations). Adding the same five mutations to the equivalent positions in the second and third quarters generated TIM2q and TIM3q, respectively (Figure 1 and Table S1). By this quarter‐wise introduction, the original four‐fold symmetry of DeNovoTIM0 and DeNovoTIM6 is broken. Therefore, the quarter variants offer interesting opportunities to investigate the effects of asymmetry, although TIM2q could still be considered pseudo‐two‐fold symmetric. All constructs were characterized in‐depth in terms of their biophysical and thermodynamic properties.

### 2.2 TIM quarter variants change their biophysical and thermodynamic properties in a stepwise fashion

**FIGURE 2:**
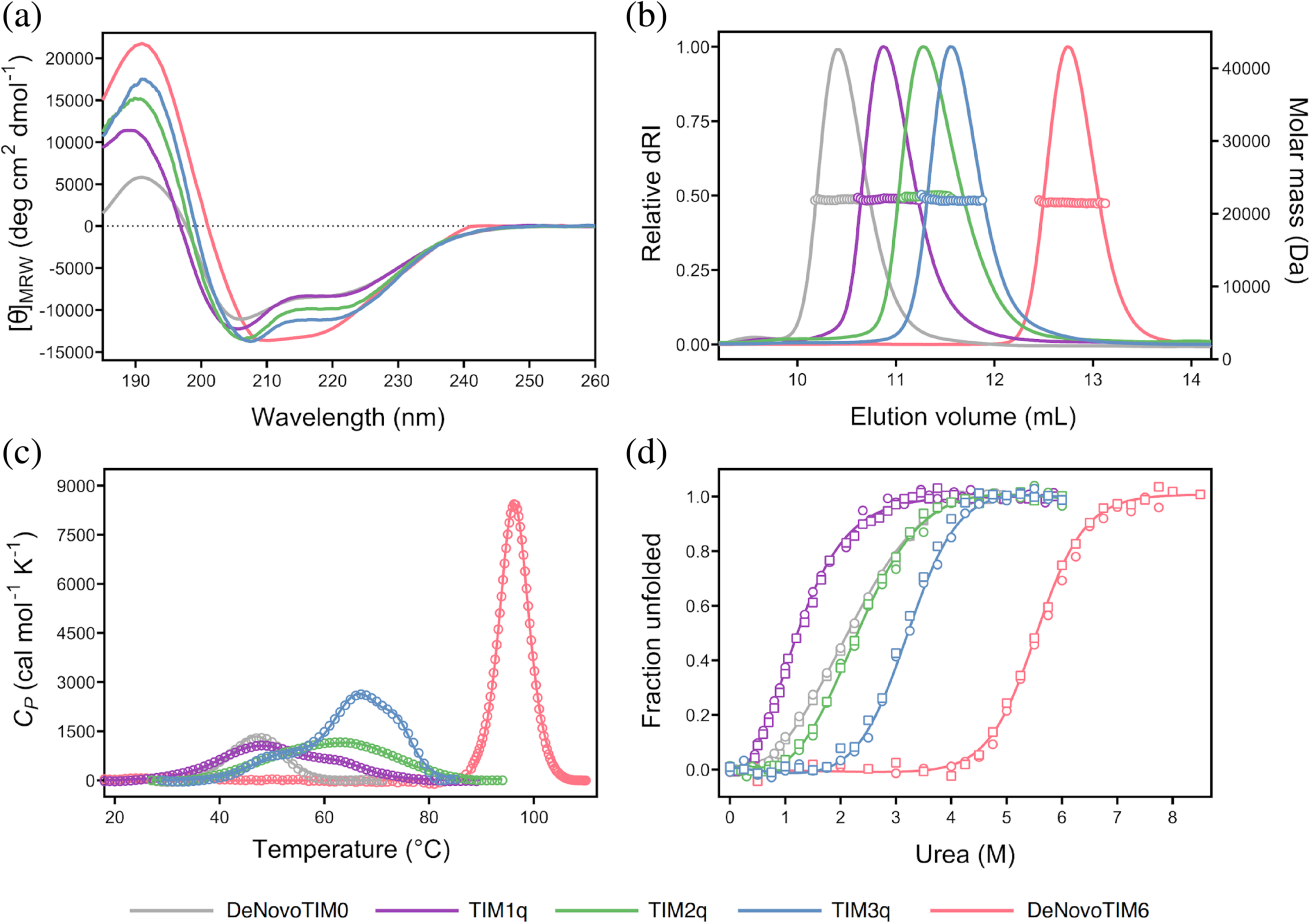
Biophysical characterization of quarter variants TIM1q, TIM2q, and TIM3q in comparison to DeNovoTIM0 and DeNovoTIM6. (a) Far‐UV CD spectra. (b) SEC‐multi angle light scattering (MALS) elution profiles depicting the signal of the refractive index (dRI, solid line) and the molar mass (empty circles). (c) Thermal unfolding by differential scanning calorimetry (DSC) showing the experimental values (open circles) and the fitting curve to the respective model (solid line) (physical and chemical baselines were subtracted for easy comparison). (d) Urea chemical‐induced unfolding followed by CD (open circles) and IF (open squares); the solid line shows the best fit to a two‐state reversible model. CD, circular dichroism; IF, intrinsic fluorescence; SEC, size exclusion chromatography.

All proteins in this study could be found in the soluble fraction upon expression in *E. coli* and were purified to homogeneity. All proteins, except DeNovoTIM6, eluted during size exclusion chromatography (SEC) in one single peak, indicating a pure and homogeneous protein preparation, corresponding to a monomeric protein as was determined in subsequent SEC‐multi‐angle light scattering (MALS) analysis (see below). DeNovoTIM6 as already shown (Romero‐Romero et al., 2021a), displayed two distinct peaks of which the first is due to dimerization. For downstream characterization, only the peak fractions containing the monomeric protein were used. Assessment of the protein's secondary structure by far‐UV circular dichroism (CD) measurements (Figure 2a) indicates the adoption of a mixture of α‐helical and β‐sheet secondary structural elements as observed for other members of the DeNovoTIM family and comparable to a TIM‐barrel topology. Interestingly, changes in the prediction of secondary structure elements (Table S2) and spectral properties, for example, location of extrema and signal intensity, can be correlated to the number of quarters containing the stabilizing mutations. These effects are even more apparent in the SEC‐MALS elution profiles (Figure 2b). From zero (DeNovoTIM0) to four (DeNovoTIM6) quarters containing stabilizing mutations, the elution peak is shifting stepwise to a higher elution volume, although not in a linear fashion. SEC is a relative and hydrodynamic technique by which molecules in solution get separated by their size and shape (La Verde et al., 2017). MALS on the other hand is an absolute method to reliably determine a protein's molar mass and oligomeric state (Some et al., 2019). Since the molar mass of all analyzed DeNovoTIMs is around 22 kDa (Table 1) and all proteins were found to be monomeric in SEC‐MALS analysis, the difference in the elution volume can be correlated to an increasingly tighter packing, resulting in higher protein compactness while more quarters contain stabilizing mutations. It should be noted that for DeNovoTIM6, although monomer and dimer populations were observed during purification (preparative SEC), only the monomer fractions were used for further characterization. Re‐dimerization of DeNovoTIM6 was ruled out based on SEC‐MALS analysis of the pooled monomeric fractions, which did not show any dimer population even after re‐loading the same sample but instead remained in the monomeric state.

**TABLE 1:** Biochemical and thermodynamic properties of the *de novo* TIM quarter variants.

	SEC‐MALS		Thermal unfolding (DSC)			Chemical unfolding (CD and IF)		
Protein	MW_theor_ (kDa)	MW_exp_ (kDa)	*T*_m_ (°C)		Δ*H*_total_ (kcal mol^−1^)	*D*_1/2_ (M)	*m* (kcal mol^−1^ M^−1^)	Δ*G* (kcal mol^−1^)
DeNovoTIM0	22.5	22.1 ± 0.2	47.0		24.7 ± 0.5	2.1	0.76 ± 0.02	1.5 ± 0.1
TIM1q	22.4	22.0 ± 0.3	*T*_m1_	46.5	27.5 ± 0.2	1.2	0.81 ± 0.06	1.0 ± 0.1
			*T*_m2_	61.1				
TIM2q	22.4	22.9 ± 0.5	*T*_m1_	53.8	34.1 ± 1.0	2.3	0.74 ± 0.13	1.9 ± 0.1
			*T*_m2_	66.7				
TIM3q	22.4	21.9 ± 0.3	*T*_m1_	51.7	54.5 ± 2.1	3.2	1.10 ± 0.10	3.8 ± 0.2
			*T*_m2_	66.2				
			*T*_m1_	73.7				
DeNovoTIM6	22.4	21.6 ± 0.1	92.3		124.9 ± 1.5	5.6	1.51 ± 0.08	7.9 ± 0.2
TIM2q‐in	22.4	21.8 ± 0.1	*T*_m1_	63.2	38.5 ± 0.3	2.4	0.90 ± 0.15	2.3 ± 0.1
			*T*_m2_	71.5				
TIM2q‐out	22.4	22.0 ± 0.3	*T*_m1_	55.5	49.8 ± 1.6	2.0	1.33 ± 0.19	2.7 ± 0.1
			*T*_m2_	64.8				

*Note*: MW_exp_ is the experimental molecular weight average of three SEC‐MALS measurements and Δ*H*_total_ is the average of five experiments at different protein concentrations. ± for SEC‐MALS and DSC indicates the standard deviation calculated for each experimental dataset while ± for chemical unfolding is the global error of fitting.

Abbreviations: CD, circular dichroism; DSC, differential scanning calorimetry; IF, intrinsic fluorescence; SEC‐MALS, size exclusion chromatography‐multi‐angle light scattering.

A similar trend of stepwise changes was observed in the thermal‐induced unfolding experiments with differential scanning calorimetry (DSC). The quarter‐wise introduction of stabilizing mutations significantly impacted the thermal‐induced unfolding process. For all initial quarter variants, the unfolding endotherm showed two (TIM1q, TIM2q) or even three transitions (TIM3q) (Figure 2c and Figure S1). DeNovoTIM0 and DeNovoTIM6 follow a two‐state process (N ⇌ U) in which only the native (N) and unfolded (U) states are populated. The quarter variants, on the other hand, unfold in a three‐ (N ⇌ I ⇌ U for TIM1q and TIM2q) or four‐state process (N ⇌ I_A_ ⇌ I_B_ ⇌ U for TIM3q) populating also long‐lived intermediate (I) states which unfold at distinct temperatures. Comparing the melting temperature (*T*_m_) values (Table 1), it should be noted that these are shifting to higher temperatures but all three variants share a transition point between 61 and 66°C. It remains to be addressed if this shared transition point might correspond to a similar unfolding intermediate. Similarly, the insertion of stabilizing mutations for one quarter (TIM1q) led to a slight increase in total unfolding enthalpy (Δ*H*_total_) compared to DeNovoTIM0 (ΔΔ*H* = 2.8 kcal mol^−1^), while for the other quarter variants, TIM2q and TIM3q, the ΔΔ*H* are considerably higher with 9.4 and 29.8 kcal mol^−1^, respectively (Figure S2a). However, the maximum change is observed from DeNovoTIM0 to DeNovoTIM6 with an increase of 100 kcal mol^−1^ in Δ*H*_total_ accompanied by a shift in the *T*_m_ from 47 to 92°C, showing that the stability increased considerably.

Furthermore, in all variants except TIM1q the quarter‐wise addition of mutations causes a similar behavior in the conformational stability in comparison to the changes in thermostability. For all investigated *de novo* TIM barrels, chemical unfolding measurements by urea revealed monophasic, cooperative, and coincident transitions (by CD and IF) that were well‐fitted to a two‐state unfolding process (Figure 2d). Based on DeNovoTIM0, the introduction of the mutations in one or two quarters (TIM1q and TIM2q) led only to slight changes in Δ*G*. The Δ*G* of TIM1q decreases by 0.5 kcal mol^−1^ while the one of TIM2q increases by 0.4 kcal mol^−1^ (Table 1 and Figure S2b). The denaturation data of TIM1q is difficult to fit due to unfolding being induced already at low urea concentrations. Increasing the number of stabilized quarters to three (TIM3q) and four (DeNovoTIM6), on the other hand, causes larger shifts in the unfolding free energy with a ΔΔ*G* of 2.3 and 6.4 kcal mol^−1^, respectively. In line with the observed increment in Δ*G*, *m* value changes followed a similar stepwise increase. Since the *m* value is a parameter that is expected to be proportional to the hydrophobic surface area (ΔASA) exposed to the solvent when a native protein is unfolded (Myers et al., 1995; Schellman, 1978; Shortle, 1995), the observed changes in the *m* value (Table 1) suggest that differences in ΔASA exposed upon unfolding exist in the TIM quarter variants. This correlates with the observed unfolding enthalpy changes in DSC and the peak shifts in SEC‐MALS measurements when more compact conformations upon the introduction of stabilizing mutations increase both Δ*H*_total_ and the retention volume. Altogether, the stability measurements show that a quarter‐wise introduction of mutations in *de novo* TIM barrels induces a stepwise increment in thermal and conformational stability nonlinearly, indicating positive nonadditive effects. Subsequently, we explored the contributions to the stability of the same number of mutated quarters but located in different regions of the barrel.

### 2.3 Biophysical properties of TIM quarter variants are affected by an altered positioning of an equivalent number of mutated regions

**FIGURE 3:**
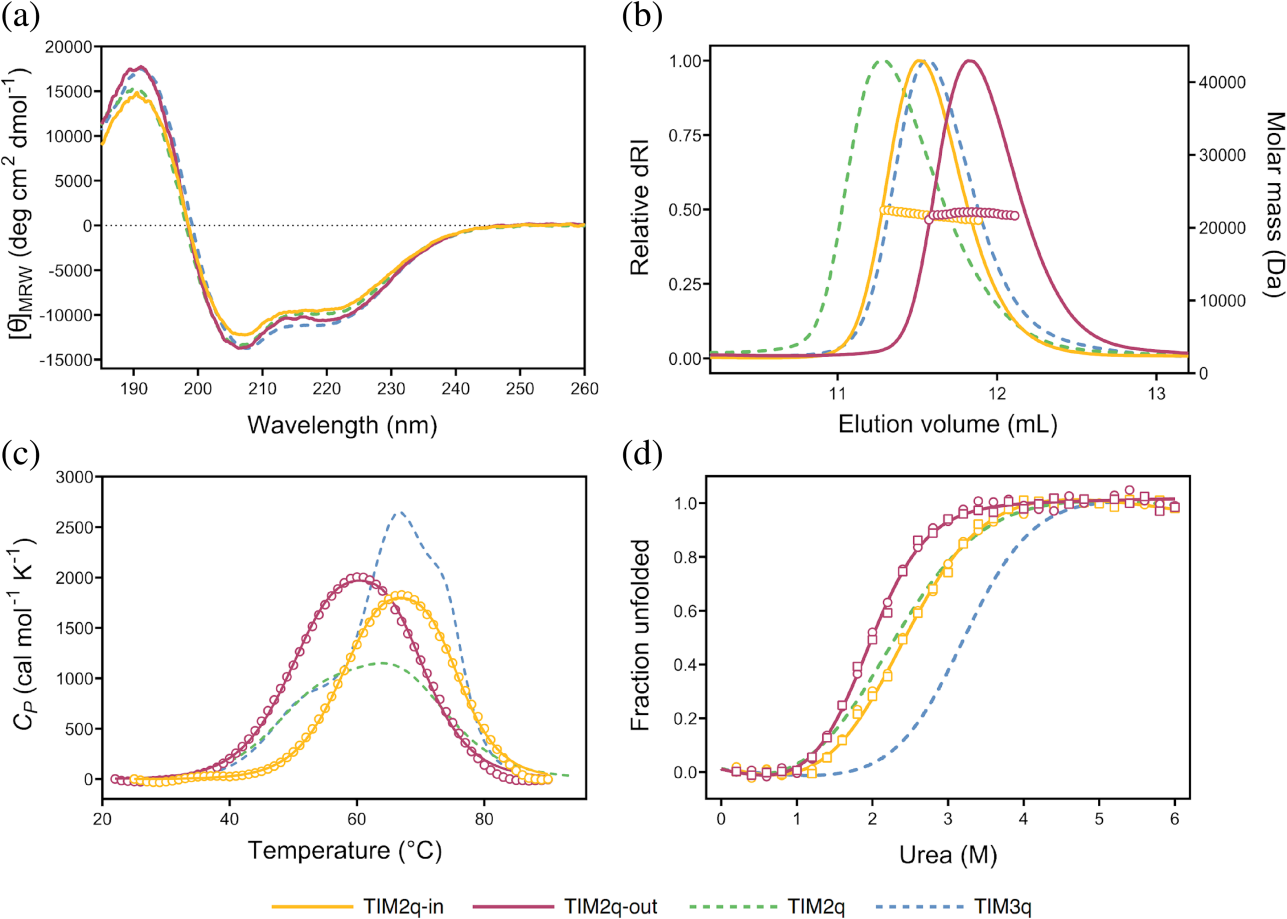
Biophysical characterization of quarter location variants TIM2q‐in and TIM2q‐out in comparison to TIM2q and TIM3q. (a) Far‐UV CD spectra. (b) SEC‐MALS elution profiles depicting the signal of the refractive index (dRI, solid line) and the molar mass (empty circles). (c) Thermal unfolding by DSC showing the experimental values (open circles) and the fitting curve to the respective model (solid line) (physical and chemical baselines were subtracted). (d) Urea chemical‐induced unfolding followed by CD (open circles) and IF (open squares); the solid line shows the best fit to a two‐state reversible model. CD, circular dichroism; DSC, differential scanning calorimetry; IF, intrinsic fluorescence; SEC‐MALS, size exclusion chromatography‐multi‐angle light scattering.

After these observations of stepwise changes in the thermodynamic and biophysical properties caused by a quarter‐wise introduction of stabilizing mutations, the question arose how these properties might be affected by varying the quarter location within the sequence instead of the number of mutated quarters. To address this, two further variants of TIM2q were designed, each with the same number of mutated quarters but different locations of the mutations. As shown in Figure 1, variant TIM2q‐in carries the stabilizing mutations in the second and third quarter which are the inner quarters in the extended amino acid sequence. Accordingly, the other variant was named TIM2q‐out since the mutations are located in the first and fourth quarters which include the N‐ and C‐terminus. Both new TIM2q variants were purified to homogeneity and underwent the same in‐depth characterization as previously discussed. Assessment of the secondary structure by far‐UV CD revealed very similar spectra compared to TIM2q and TIM3q (Figure 3a and Table S2), and in SEC‐MALS measurements the location variants displayed the expected molar mass of around 22 kDa similar to the other TIM proteins (Table 1). However, the location variants exhibited a considerable shift in their elution peak (Figure 3b). Although the same number of quarters are mutated, TIM2q‐in and TIM2q‐out seem to have a different degree of compactness compared to TIM2q since the elution volumes in SEC are quite different. Interestingly, TIM2q‐in and TIM2q‐out also show differences between each other: TIM2q‐in elutes slightly earlier than TIM3q while TIM2q‐out elutes rather later.

Variation in the quarter location did not change the unfolding process (three‐state like TIM2q) but impacted thermostability for TIM2q‐in, whose transition midpoints shifted to higher temperatures compared to TIM2q and are very similar to TIM3q (Figure 3c and Figure S1). When comparing the *T*_m_ values, the presence of a transition in the range of 61–66°C was observed as noticed for the other quarter variants. Since TIM2q‐in and TIM3q share the location of mutated quarters in the second and third quarters, it is interesting that the *T*_m_ values are also similar (Table 1). In terms of the unfolding enthalpy, ΔΔ*H*_total_ for TIM2q‐in increased only to 13.8 kcal mol^−1^ which is slightly higher than TIM2q. On the other hand, it is remarkable that TIM2q and TIM2q‐out, which have the location of mutations in the first quarter in common and both show similar *T*_m_ values, reveal differences in Δ*H*_total_ (Table 1). For TIM2q, the ΔΔ*H*_total_ was 9.4 kcal mol^−1^ but increased to 25.1 kcal mol^−1^ for TIM2q‐out (Figure S2a). The observed increases in Δ*G* and Δ*H* indicate that stabilization of the N‐ and C‐terminal quarters is especially crucial since these quarters are involved in the closure of the barrel structure, in the same way that has been described for other repetitive proteins with closed architectures (Doyle et al., 2015). Therefore, tight packing of the secondary structure elements in this region is of considerable relevance.

A similar tendency is observed in the Δ*G* determined by chemical‐induced unfolding (Figure 3d). While slight changes in the *D*_1/2_ were observed, significant changes were noticed for the *m* values (Table 1). For example, even though the *D*_1/2_ for TIM2q‐out is the lowest, a significant increase in *m* value is observed. These changes are reflected in an increase of ΔΔ*G* from 0.4 (TIM2q) to 0.8 (TIM2q‐in) or 1.2 kcal mol^−1^ (TIM2q‐out). Nevertheless, the stability in the location variants is still lower than for TIM3q. Altogether, the results emphasize the importance of the N‐ and C‐terminal quarters on the stability, specifically during the initial hydrophobic collapse for a cooperative folding process (Figure S2b). Varying the location of stabilized quarters revealed that the mutations have different impacts on the overall protein stability depending on the region they were introduced to.

### 2.4 Quarter interfaces influence the stability changes in TIM quarter variants

The stabilizing mutations in DeNovoTIM6 are mainly located in the α‐helices at positions facing the helix residues of the neighboring quarter, thereby spanning a different helix–helix interface than in DeNovoTIM0. The quarter‐wise introduction of stabilizing mutations led to a mix‐and‐match of these residues and modified helix–helix interfaces in the barrel. In general, using TIM2q as an example, there are four types of interface residues to consider when mixing DeNovoTIM0 and DeNovoTIM6 quarter by quarter (Figure S3): “*non‐stabilized/non‐stabilized*” when both quarters are from DeNovoTIM0, “*non‐stabilized/stabilized*” for interfaces with DeNovoTIM0 and DeNovoTIM6 residues, “*stabilized/non‐stabilized*” for quarters with DeNovoTIM6 and DeNovoTIM0 residues, and “*stabilized/stabilized*” interfaces when both quarters contain mutations from DeNovoTIM6.

**FIGURE 4:**
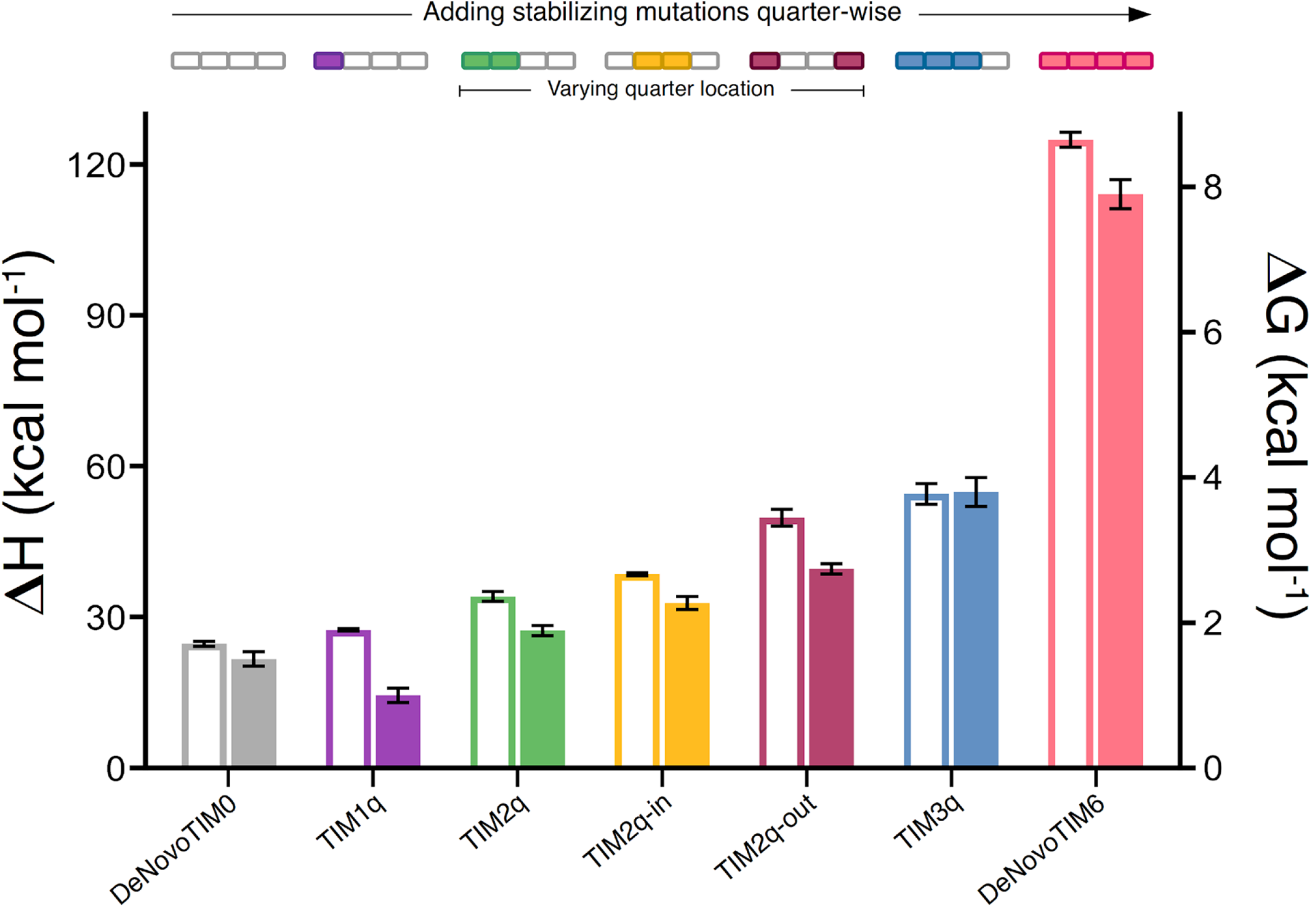
Comparative stability analysis of the investigated *de novo* TIM quarter variants. The plot shows for each protein the unfolding Δ*H* (left *y*‐axis, open bars) obtained by DSC and unfolding Δ*G* (right *y*‐axis, filled bars) determined from chemical unfolding with urea. DSC, differential scanning calorimetry.

For TIM1q, the helices carrying stabilizing mutations located in quarter 1 face quarters 4 and 2, both composed of residues from DeNovoTIM0. Thus, this variant contains three types of interfaces, that is, either “*non‐stabilized/stabilized*” and “*stabilized/non‐stabilized*” when quarter 1 is involved, or “*non‐stabilized/non‐stabilized*” between all other quarters. The *non‐stabilized/stabilized* interface specifically is suboptimal as it leads to a lysine residue in an otherwise hydrophobic interface without any compensatory interaction (top panel in Figure S3). This might explain the lower stability observed in TIM1q in comparison to all other constructs. Differently, since in TIM2q the second quarter is mutated as well, the interface between quarters 1 and 2 is similarly stabilized like in DeNovoTIM6, that is, already one “*stabilized/stabilized*” interface is present (Figure S3), which exhibits an increased hydrophobicity. These hydrophobic residues might form favorable van der Waals contacts, providing a dominant driving force for folding and stability as has been described for most proteins (Nicholls et al., 1991; Pace et al., 2011, 2014). In the TIM2q variants, TIM2q‐in and TIM2q‐out, this stabilized interface is shifted to different locations. In TIM2q‐in, it is located between the second and third quarters, and in TIM2q‐out between the first and fourth. Interestingly, the location of these mutations has an important influence on the observed stability effects, which we attribute to the closed architecture of the TIM‐barrel fold as described.

Moreover, while TIM2q has only one fully stabilized interface, TIM3q holds two such fully stabilized interfaces, namely between quarters 1 and 2, as well as 2 and 3. The observed changes in Δ*H*_total_ and Δ*G* from DeNovoTIM0 to TIM1q are rather minimal compared to variants with more stabilized quarters (TIM2q and TIM3q) (Figure 4). In addition, from TIM1q to TIM2q and then to TIM3q and DeNovoTIM6, the observed thermodynamic changes are stepwise increased but in a nonlinear fashion, suggesting that the stability effects are nonadditive. For these TIM quarter variants, since the introduced mutations are mostly hydrophobic and no notable changes in hydrogen bonds or salt bridges were observed in the models, it can be considered that the main contribution to the gained stability is due to increased van der Waals interactions, in the same way as has been described for other hydrophobic variants (Baase et al., 2010; Eriksson et al., 1992; Loladze et al., 2002; Ratnaparkhi and Varadarajan, 2000). Overall. the results suggest that the number of stabilized interfaces between quarters is a key factor in modulating the conformational stability in these four‐fold symmetric TIM barrels. This conclusion is in line with recent design approaches of symmetric and asymmetric proteins by exploiting interface‐driven strategies (Broom et al., 2012; Cao et al., 2022; ElGamacy et al., 2018; Maguire et al., 2021; Rhys et al., 2018). Analysis of the location variants already showed that stabilization of different regions with the same number of mutations, for example, shifting the location of the stabilized interface, has a considerable impact on protein stability. Testing variants in which the mutations are introduced per quarter‐interface instead of sequence quarter‐wise might provide deeper insights into the structural determinants that modulate the stability in this *de novo* TIM‐barrel family.

## 3 CONCLUSIONS

Protein stability is one of the main parameters that shape protein properties and has been a cornerstone in computational design and protein folding investigations. Here, we studied the effects of introducing stabilizing mutations step by step to a four‐fold symmetric *de novo* TIM‐barrel protein. Through a comprehensive analysis of their biophysical and thermodynamic properties, we uncovered a nonlinear increase in both thermal and conformational stability that aligns with the addition of the same five mutations per sequence quarter. Our findings suggest that the favorable and nonadditive effects on stability by quarter‐wise mutation might be modulated by alterations in protein packing and reinforced by hydrophobic stabilization at the TIM barrel interfaces. Moreover, we observed that changing the locations of the same number of mutated quarters influences the stability within this fold, highlighting the significance of achieving barrel closure in repetitive proteins with closed architectures. A deep analysis of *de novo* proteins, as exemplified in this study, enhances our understanding of how sequence variations can finely calibrate stability both in naturally occurring and computationally designed proteins.

## 4 MATERIALS AND METHODS

### 4.1 Chemicals

All reagents were analytical grade from Merck KGaA or Carl Roth. All solutions and buffers were prepared with double‐distilled water.

### 4.2 Cloning, overexpression, and protein purification

All synthetic genes of the quarter variants were obtained from BioCat cloned in the pET21b(+) vector containing a C‐terminal His‐tag. DeNovoTIM0 and DeNovoTIM6 are instead cloned in pET29b(+) as described in Romero‐Romero et al. (2021a). LB precultures (with either 100 μg mL^−1^ ampicillin or 50 μg mL^−1^ kanamycin) were inoculated with transformed *E. coli* BL21(DE3) and grown overnight at 37°C and 180 rpm. One liter of Terrific Broth (TB) media (with either 100 μg mL^−1^ ampicillin or 50 μg mL^−1^ kanamycin) was inoculated at approximately OD_600_: 0.08 and incubated at 37°C and 180 rpm until optical density reached OD_600_: 0.6. Then, protein overexpression was induced using 1 mM isopropyl‐d‐1‐thiogalactopyranoside. Incubation was continued for 4 h at 30°C and 180 rpm. Subsequently, cells were harvested by centrifugation (Beckmann Avanti JLA‐8.1000, 12 min, 5000 rpm, 4°C) followed by pellet resuspension in buffer A (35 mM sodium phosphate, 300 mM NaCl, 35 mM imidazole pH 8.0) supplemented with 200 μL protease inhibitor (Mix‐HP, Serva) per 5 g of pellet. Cell lysis was performed by sonication (Branson Ultrasonics) (4 times 2 min, output 4, duty cycle 40%). Afterward, the lysate was clarified by centrifugation (Beckmann Avanti JA‐25.50, 1 h, 18,000 rpm, 4°C) and filtered (0.22 μm filter by Merck Millipore) prior to loading the lysate onto an equilibrated (buffer A) HisTrap FF column (Cytiva Life Sciences) which was coupled to an ÄKTA pure™ protein purification system (GE Healthcare Life Sciences). A washing step with 20 column volumes (CV) buffer A was performed to remove unbound proteins followed by protein elution by application of a linear gradient of 35–500 mM imidazole using buffer B (35 mM sodium phosphate, 300 mM NaCl, 500 mM imidazole pH 8.0). Peak fractions were pooled and concentrated for subsequent purification via SEC. For this, a HiLoad 26/600 Superdex 75 preparative grade column (GE Healthcare Life Sciences) connected to an ÄKTA pure™ protein purification system was used. Protein was eluted using isocratic elution with 1 CV of buffer C (35 mM sodium phosphate, 150 mM NaCl pH 8.0), and the peak fractions were analyzed by SDS‐PAGE. Pure monomeric fractions were pooled, concentrated, and stored at 4°C for immediate experiments or at −20°C for further use. Some downstream analysis required the protein to be dialyzed into buffer D (10 mM sodium phosphate, pH 8.0) using a dialysis membrane (3.5 kDa MWCO) and performing multiple buffer changes at 4°C while stirring.

### 4.3 Far‐UV CD

All CD measurements were performed using a Jasco J‐710 spectropolarimeter connected to a Peltier device (PTC‐348 WI) for temperature control. Far‐UV CD spectra were collected using a protein concentration of 0.2 mg mL^−1^ in buffer D in a 0.2 cm cuvette. The temperature was set to 25°C and a wavelength range of 185–260 nm was chosen. Data normalization was performed by subtraction of buffer spectra and subsequent conversion of the measured signal in mdeg to mean residue molar ellipticity by: [*θ*_MRW_] = (*M*·*θ*)/(10·*d*·*c*) and *M* = (MW/(*n* − 1)) (Greenfield, 2006), where *c* is protein concentration in mg mL^−1^, *d* the path length in cm, *θ* the measured signal in mdeg, *M* the mean residue weight, MW the molecular weight in g mol^−1^, and *n* is the number of residues in the protein. Far‐UV spectra were deconvoluted with the BeStSel webserver to obtain the predicted composition of secondary structure elements (Micsonai et al., 2022).

### 4.4 Intrinsic fluorescence

Intrinsic fluorescence (IF) spectra were measured at 0.2 mg mL^−1^ protein concentration in buffer D. Measurements were performed with a Jasco FP‐6500 spectrofluorometer connected to a Peltier device (Julabo MB) for temperature control. Fluorescence emission spectra were measured in the wavelength range of 310–450 nm after excitation at 280 and 295 nm with a bandwidth of 1 nm. The spectral center of mass (SCM) was calculated from intensity data (*I*_*λ*_) collected at different wavelengths (*λ*) by SCM = ∑*λI*_*λ*_/∑*I*_*λ*_.

### 4.5 MALS coupled to a SEC

For SEC‐MALS measurements, a Superdex 75 Increase 10/300 GL column (Cytiva Life Sciences) coupled to an ÄKTA pure™ protein purification system was used, which was further connected to a miniDAWN MALS detector and an Optilab differential refractive index (dRI) detector (Wyatt Technology). Measurements were performed at room temperature using buffer C with the addition of 0.02% sodium azide, 0.8 mL min^−1^ flow rate, and protein concentrations of 1, 2, and 3 mg mL^−1^ with an injection volume of 100 μL. Reproducibility of the SEC‐MALS runs was ensured by obtaining identical results in the measured BSA (bovine serum albumin) samples (Pierce) at 2 mg mL^−1^ as the first and last sample of each day's measurements. The first BSA injection was used for detector normalization, peak alignment, and band broadening. The dRI signal was used as a concentration source for molar mass determination in all cases. Data were collected and analyzed using ASTRA 8.0.2.5 software (Wyatt Technology).

### 4.6 Thermal unfolding followed by DSC

DSC was employed to measure the temperature‐induced unfolding process using a Microcal PEAQ‐DSC (Malvern Panalytical). Samples were assayed with a scan rate of 90 K h^−1^ covering a temperature range of 10–120°C and protein concentration of 1, 2, 3, 4, and 5 mg mL^−1^ in buffer D. The buffer was extensively degassed prior to use in all DSC runs. Prior to sample‐buffer scans, multiple buffer‐buffer scans were performed for equilibration. For the thermodynamics analysis, the precedent buffer‐buffer scan was subtracted from the sample‐buffer scan. Reversibility was examined for each protein by immediate measurement of a second endotherm after cooling down from the first measurement, resulting in a reversible process for all proteins. Since DeNovoTIM0 and DeNovoTIM6 followed a two‐state reversible unfolding process, the endotherms were fitted to a two‐state model (Equation 1) (Romero‐Romero et al., 2015, 2021a):

(1) $$C_{P}\left(T\right)=B_{0}+B_{1}T+f\left(T\right)\Delta C_{P}+\frac{\Delta H\left(T\right)}{RT_{m}^{2}}\left[\frac{1-f\left(T\right)}{1-n+\frac{n}{f\left(T\right)}}\right],$$

where *B*_0_ and *B*_1_ represent pre‐ and post‐transition constants, respectively, *f*(*T*) is the protein fraction in the folded monomeric state, *n* represents the number of subunits of the native protein (monomer for all proteins examined), yielding the fitting parameters Δ*H*, Δ*C*_P_, and *T*_m_. For all TIM quarter variants (TIM1q, TIM2q, TIM3q, TIM2q‐in, and TIM2q‐out) a two‐state model did not fit the experimental data well and more transitions were needed to gain a proper fitting curve. Therefore, endotherms were analyzed using a non‐two‐state model with independent transitions. Because this model only applies to transitions with no Δ*C*_P_, the progress chemical baseline was subtracted from the experimental data to remove Δ*C*_P_ if they were present. All the calorimetric transitions were fitted according to Equations (2) and (3) (Microcal PEAQ‐DSC manual):

(2) $$C_{P}\left(T\right)=\frac{K_{A}\left(T\right)\Delta H^{2}}{{\left[1+K_{A}\left(T\right)\right]}^{2}RT^{2}}+\cdots ,$$

(3) $$K_{A}\left(T\right)=\exp\left\{\left[\frac{-\Delta H}{\mathrm{RT}}\right]\times \left(1-\frac{T}{T_{m}}\right)\right\},$$

where *T* is the temperature at each data point, *K*_A_ is the equilibrium constant, Δ*H* is the van't Hoff enthalpy (corresponding to the enthalpy change for the cooperative unit which actually participates in each transition), *R* is the universal gas constant, and *T*_m_ is the melting temperature at the maximum *C*_P_ for each independent transition (2 for TIM1q, TIM2q, TIM2q‐in, and TIM2q‐out, and 3 for TIM3q). Calorimetric data were processed and fitted using the MicroCal PEAQ‐DSC software and DSC Data Analysis guide.

### 4.7 Chemical‐induced unfolding followed by CD and IF

Samples of all variants were prepared at 150 μg mL^−1^ in buffer D with urea concentrations ranging between 0 and 6.0 M (except for DeNovoTIM6 which ranged up to 9 M) and incubated overnight at 25°C. Since previous chemical‐induced unfolding experiments on the DeNovoTIM family (Romero‐Romero et al., 2021a) showed that at least 12 h of incubation are sufficient to reach equilibrium, the same incubation period was applied here. The CD signal was followed for 120 s at 222 nm for each sample and data were pre‐processed by calculating the average CD signal for each sample. IF measurements were performed as described and data were pre‐processed by calculating the SCM for each sample. Both, CD and IF data were then normalized to the fraction of unfolded molecules by (Equation 4):

(4) $$f_{U}=\frac{y_{\mathrm{obs}}-\left(y_{N}+m_{N}\left[\text{urea}\right]\right)\;}{\left(y_{U}+m_{U}\left[\text{urea}\right]\right)-\left(y_{N}+m_{N}\left[\text{urea}\right]\right)},$$

where (*y*_N_ + *m*_N_[urea]) and (*y*_U_ + *m*_U_[urea]) represent the linear fitting equations of the native and unfolded regions, respectively and *y*_obs_ is the observed experimental CD or IF signal at a given urea concentration. Determination of the unfolding free energy Δ*G* was performed by a global fitting of the CD and IF data to a two‐state model (N ⇌ D) using the Santoro and Bolen equation (Equation 5) (Santoro and Bolen, 1988):

(5) $$f_{U}=\frac{\left(y_{N}+m_{N}\left[\text{urea}\right]\right)+\left(y_{U}+m_{U}\left[\text{urea}\right]\right)\times e^{-\frac{\Delta G-m\left[\text{urea}\right]}{R\;T}}}{1+e^{-\frac{\Delta G-m\left[\text{urea}\right]}{R\;T}}},$$

where Δ*G* is the unfolding free energy in absence of denaturant, *m* the dependence of Δ*G* to the denaturant (Δ*G*/[urea]), *T* the temperature of the experiment (298.15 K), *R* the universal gas constant, and (*y*_N_ + *m*_N_[urea]) and (*y*_U_ + *m*_U_[urea]) represent the linear fitting equations of the pre‐ and post‐transition baselines (folded and unfolded states), respectively. *D*_1/2_ was calculated by using the nonlinear fitting curve values and determining the corresponding urea value where 50% of native and unfolded fractions are present. Data processing and fitting were performed with Origin v.9.0 (OriginLab Corporation, Northampton, MA, USA).

## AUTHOR CONTRIBUTIONS

**Johanna‐Sophie Koch:** Investigation; writing – original draft; writing – review and editing; formal analysis; methodology. **Sergio Romero Romero:** Conceptualization; investigation; writing – original draft; methodology; validation; visualization; writing – review and editing; formal analysis; supervision; data curation. **Birte Höcker:** Conceptualization; funding acquisition; writing – original draft; writing – review and editing; project administration; supervision; resources; formal analysis; methodology.

## FUNDING INFORMATION

This work was supported by the European Research Council (ERC Consolidator Grant 647548 to B.H.), the VolkswagenStiftung (grant 94747 to B.H.), and a fellowship from the Alexander von Humboldt and Bayer Science & Education Foundations (Humboldt‐Bayer Research Fellowship for Postdoctoral Researchers to S.R.R.).

## CONFLICT OF INTEREST STATEMENT

The authors declare no conflicts of interest.

## Supporting information


**Figure S1.** DSC fitting curves of the *de novo* TIM quarter variants. Each plot shows the experimental data (open circles), the overall fitting curve (solid line), as well as the fitting curves for individual peaks (dashed lines). (a) TIM1q in purple (——), (b) TIM2q in green (——), (c) TIM2q‐in in yellow (——), (d) TIM2q‐out in red (——), and (e) TIM3q in blue (——).
**Figure S2.** Changes in (a) total unfolding enthalpy (ΔΔH) and (b) unfolding free energy (ΔΔG) of the *de novo* TIM quarter variants. All changes were calculated using DeNovoTIM0 as a reference. Actual values are shown above each symbol.
**Figure S3.** Different types of interfaces upon introduction of stabilizing mutations in the *de novo* TIM barrel quarters. The TIM2q model is shown for explanatory purposes. For better visualization, quarter one is shown in the color of TIM1q (purple) and quarter two is shown in the color of TIM2q (green). Since quarters three and four correspond sequence‐wise to DeNovoTIM0, they are shown in gray. Mutated residues in TIM2q and the corresponding (non‐mutated) residues in DeNovoTIM0 are highlighted as sticks. Quarter 4 and 1 interface corresponds to the interface between “non‐stabilizing” residues (from DeNovoTIM0) and “stabilizing residues” (from DeNovoTIM6). Quarter 1 and 2 interface contains two quarters with stabilizing residues. Quarter 2 and 3 interface conforms to the interface between a stabilized quarter and a non‐stabilized one. Finally, the interface of quarters 3 and 4 shows the interface between two non‐stabilized quarters.
**Table S1.** Amino acid sequences of all analyzed *de novo* TIM quarter variants. Positions of the stabilizing mutations are highlighted in bold and red.
**Table S2.** Predicted secondary structure content by CD spectra deconvolution. Native far‐UV CD spectra were analyzed with the BeStSel webserver (Micsonai et al., 2022).

## Data Availability

All data to support the conclusions of this manuscript are included in the main text and supplementary information.
