# Supplementary material for: Stepwise introduction of stabilizing mutations reveals nonlinear additive effects in de novo TIM barrels
Source: Protein Sci. 2024 Feb 21;33(3):e4926. doi: 10.1002/pro.4926 (PMC10880431; doi:10.1002/pro.4926)
Supplement: Supplementary file 1 — Figure S1. DSC fitting curves of the de novo TIM quarter variants. Each plot shows the experimental data (open circles), the overall fitting curve (solid line), as well as the fitting curves for individual peaks (dashed lines). (a) TIM1q in purple (——), (b) TIM2q in green (——), (c) TIM2q‐in in yellow (——), (d) TIM2q‐out in red (——), and (e) TIM3q in blue (——). Figure S2. Changes in (a) total unfolding enthalpy (ΔΔH) and (b) unfolding free energy (ΔΔG) of the de novo TIM quarter variants. All changes were calculated using DeNovoTIM0 as a reference. Actual values are shown above each symbol. Figure S3. Different types of interfaces upon introduction of stabilizing mutations in the de novo TIM barrel quarters. The TIM2q model is shown for explanatory purposes. For better visualization, quarter one is shown in the color of TIM1q (purple) and quarter two is shown in the color of TIM2q (green). Since quarters three and four correspond sequence‐wise to DeNovoTIM0, they are shown in gray. Mutated residues in TIM2q and the corresponding (non‐mutated) residues in DeNovoTIM0 are highlighted as sticks. Quarter 4 and 1 interface corresponds to the interface between “non‐stabilizing” residues (from DeNovoTIM0) and “stabilizing residues” (from DeNovoTIM6). Quarter 1 and 2 interface contains two quarters with stabilizing residues. Quarter 2 and 3 interface conforms to the interface between a stabilized quarter and a non‐stabilized one. Finally, the interface of quarters 3 and 4 shows the interface between two non‐stabilized quarters. Table S1. Amino acid sequences of all analyzed de novo TIM quarter variants. Positions of the stabilizing mutations are highlighted in bold and red. Table S2. Predicted secondary structure content by CD spectra deconvolution. Native far‐UV CD spectra were analyzed with the BeStSel webserver (Micsonai et al., 2022). [file PRO-33-e4926-s001.docx]

# Supporting information

**Step-wise introduction of stabilizing mutations reveals nonlinear additive effects in *de novo* TIM barrels**

Johanna-Sophie Koch,^1^ Sergio Romero-Romero,^1*^ Birte Höcker^1*^

^1^ Department of Biochemistry, University of Bayreuth, 95447 Bayreuth, Germany.

* Corresponding authors:

Sergio Romero-Romero. e-mail address: [sergio.romero-romero@uni-bayreuth.de](mailto:sergio.romero-romero@uni-bayreuth.de)

Birte Höcker. e-mail address: [birte.hoecker@uni-bayreuth.de](mailto:birte.hoecker@uni-bayreuth.de)

This file includes:

- Supplementary Figures 1-3.
- Supplementary Tables 1-2.

# Supplementary figures


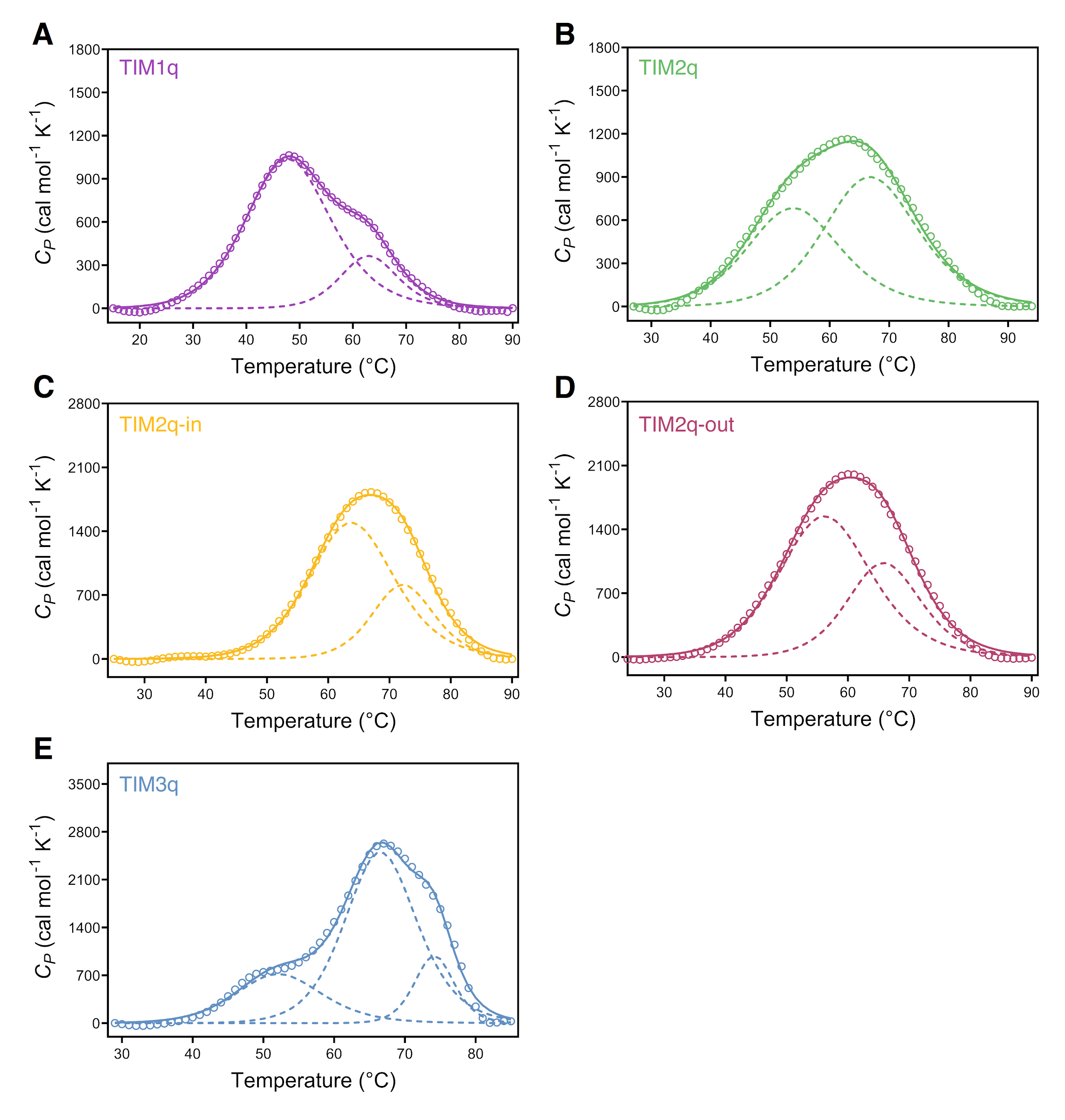


**Supplementary Figure S1.** **DSC fitting curves of the *de novo* TIM quarter variants**. Each plot shows the experimental data (open circles), the overall fitting curve (solid line), as well as the fitting curves for individual peaks (dashed lines). (A) TIM1q in purple (——), (B) TIM2q in green (——), (C) TIM2q-in in yellow (——), (D) TIM2q-out in red (——), and (E) TIM3q in blue (——).


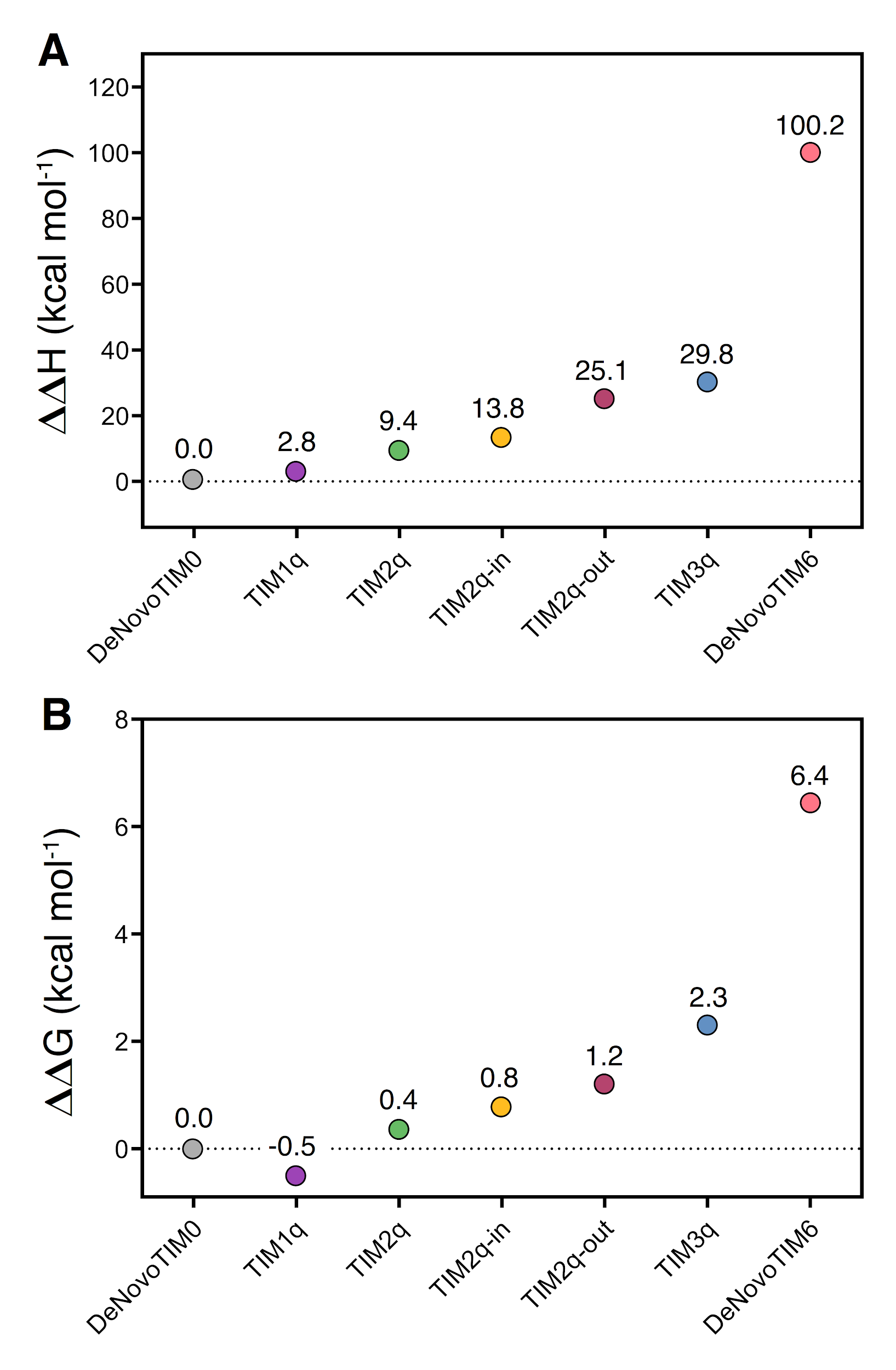


**Supplementary Figure S2.** **Changes in (A) total unfolding enthalpy (**𝚫𝚫**H) and (B) unfolding free energy (**𝚫𝚫**G) of the *de novo* TIM quarter variants**. All changes were calculated using DeNovoTIM0 as a reference. Actual values are shown above each symbol.


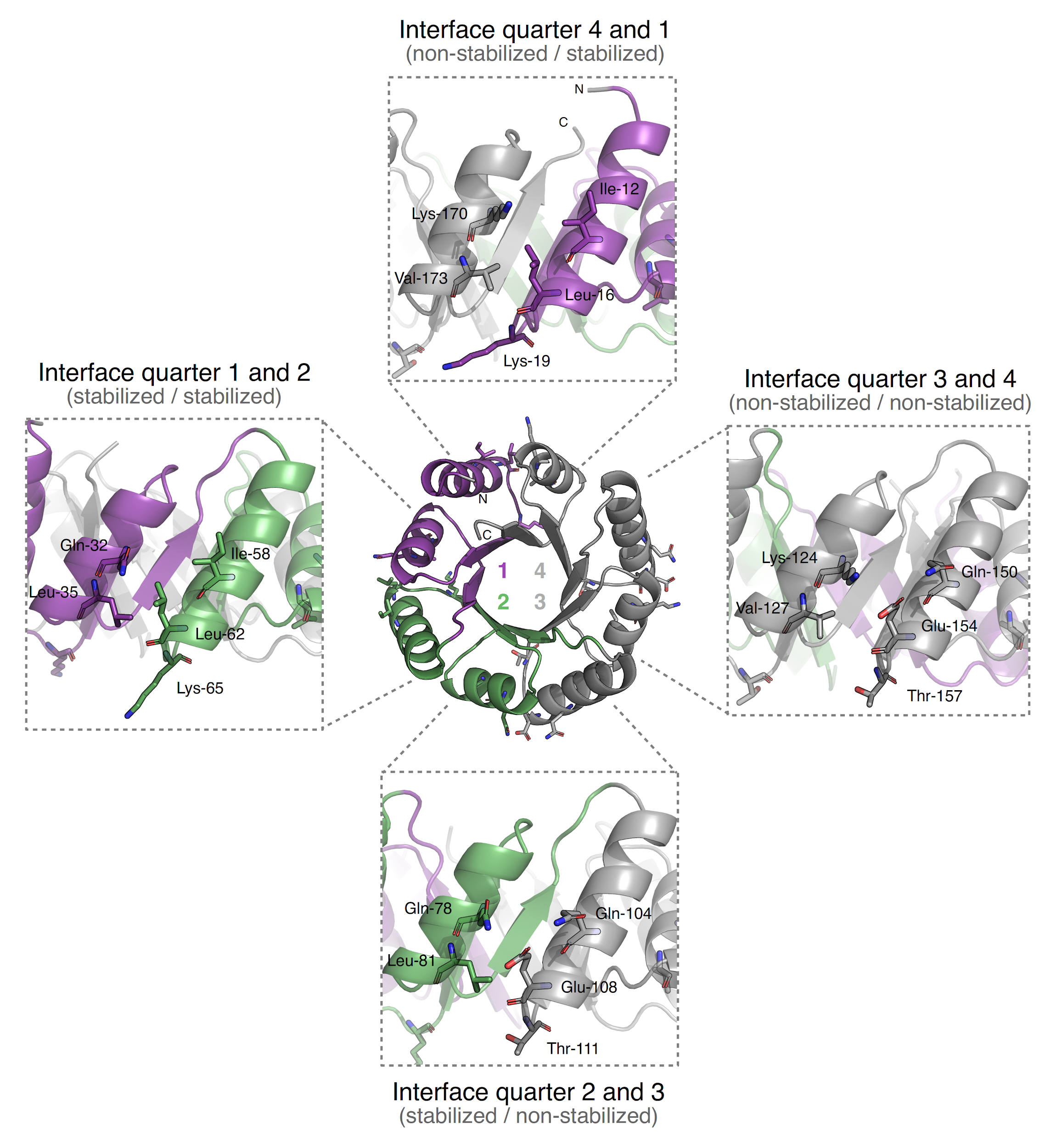


**Supplementary Figure S3.** **Different types of interfaces upon introduction of stabilizing mutations in the *de novo* TIM barrel quarters**. The TIM2q model is shown for explanatory purposes. For better visualization, quarter one is shown in the color of TIM1q (purple) and quarter two is shown in the color of TIM2q (green). Since quarters three and four correspond sequence-wise to DeNovoTIM0, they are shown in gray. Mutated residues in TIM2q and the corresponding (non-mutated) residues in DeNovoTIM0 are highlighted as sticks. Quarter 4 and 1 interface corresponds to the interface between “non-stabilizing” residues (from DeNovoTIM0) and “stabilizing residues” (from DeNovoTIM6). Quarter 1 & 2 interface contains two quarters with stabilizing residues. Quarter 2 & 3 interface conforms to the interface between a stabilized quarter and a non-stabilized one. Finally, the interface of quarters 3 and 4 shows the interface between two non-stabilized quarters.

# Supplementary tables

**Supplementary Table S1.** **Amino acid sequences of all analyzed *de novo* TIM quarter variants**. Positions of the stabilizing mutations are highlighted in bold and red.

| **Protein** | **Sequence** |
| --- | --- |
| **DeNovoTIM0** | MDKDEAWKQVEQLRREGATQIAYRSDDWRDLKEAVKKGGDILIVDAT  DKDEAWKQVEQLRREGATQIAYRSDDWRDLKEAVKKGGDILIVDAT  DKDEAWKQVEQLRREGATQIAYRSDDWRDLKEAVKKGGDILIVDAT  DKDEAWKQVEQLRREGATQIAYRSDDWRDLKEAVKKGGDILIVDAT  LEHHHHHH |
| **TIM1q** | MDKDEAWKQVE**I**LRR**L**GA**K**QIAYRSDDWRDL**Q**EA**L**KKGGDILIVDAT  DKDEAWKQVEQLRREGATQIAYRSDDWRDLKEAVKKGGDILIVDAT  DKDEAWKQVEQLRREGATQIAYRSDDWRDLKEAVKKGGDILIVDAT  DKDEAWKQVEQLRREGATQIAYRSDDWRDLKEAVKKGGDILIVDAT  LEHHHHHH |
|  |  |
| **TIM2q** | MDKDEAWKQVE**I**LRR**L**GA**K**QIAYRSDDWRDL**Q**EA**L**KKGGDILIVDAT  DKDEAWKQVE**I**LRR**L**GA**K**QIAYRSDDWRDL**Q**EA**L**KKGGDILIVDAT  DKDEAWKQVEQLRREGATQIAYRSDDWRDLKEAVKKGGDILIVDAT  DKDEAWKQVEQLRREGATQIAYRSDDWRDLKEAVKKGGDILIVDAT  LEHHHHHH |
|  |  |
| **TIM3q** | MDKDEAWKQVE**I**LRR**L**GA**K**QIAYRSDDWRDL**Q**EA**L**KKGGDILIVDAT  DKDEAWKQVE**I**LRR**L**GA**K**QIAYRSDDWRDL**Q**EA**L**KKGGDILIVDAT  DKDEAWKQVE**I**LRR**L**GA**K**QIAYRSDDWRDL**Q**EA**L**KKGGDILIVDAT  DKDEAWKQVEQLRREGATQIAYRSDDWRDLKEAVKKGGDILIVDAT  LEHHHHHH |
|  |  |
|  |  |
| **DeNovoTIM6** | MDKDEAWKQVE**I**LRR**L**GA**K**QIAYRSDDWRDL**Q**EA**L**KKGGDILIVDAT  DKDEAWKQVE**I**LRR**L**GA**K**QIAYRSDDWRDL**Q**EA**L**KKGGDILIVDAT  DKDEAWKQVE**I**LRR**L**GA**K**QIAYRSDDWRDL**Q**EA**L**KKGGDILIVDAT  DKDEAWKQVE**I**LRR**L**GA**K**QIAYRSDDWRDL**Q**EA**L**KKGGDILIVDAT  GLEHHHHHH |
| **TIM2q-in** | MDKDEAWKQVEQLRREGATQIAYRSDDWRDLKEAVKKGGDILIVDAT  DKDEAWKQVE**I**LRR**L**GA**K**QIAYRSDDWRDL**Q**EA**L**KKGGDILIVDAT  DKDEAWKQVE**I**LRR**L**GA**K**QIAYRSDDWRDL**Q**EA**L**KKGGDILIVDAT  DKDEAWKQVEQLRREGATQIAYRSDDWRDLKEAVKKGGDILIVDAT  LEHHHHHH |
|  |  |
| **TIM2q-out** | MDKDEAWKQVE**I**LRR**L**GA**K**QIAYRSDDWRDL**Q**EA**L**KKGGDILIVDAT  DKDEAWKQVEQLRREGATQIAYRSDDWRDLKEAVKKGGDILIVDAT  DKDEAWKQVEQLRREGATQIAYRSDDWRDLKEAVKKGGDILIVDAT  DKDEAWKQVE**I**LRR**L**GA**K**QIAYRSDDWRDL**Q**EA**L**KKGGDILIVDAT  LEHHHHHH |
|  |  |

**Supplementary Table S2.** **Predicted secondary structure content by CD spectra deconvolution.** Native far-UV CD spectra were analyzed with the BeStSel webserver [(Micsonai et al. 2022)](https://www.zotero.org/google-docs/?QRapXa).

| **Protein** | **Predicted secondary structure content (%)** | | | | |
| --- | --- | --- | --- | --- | --- |
|  | **ɑ-helix** | **β-strand** | **Turn** | **Random coil/Other** | **Total sum** |
| **DeNovoTIM0** | 17.7 | 21.5 | 13.3 | 47.0 | 100 |
| **TIM1q** | 18.3 | 19.4 | 15.7 | 46.3 | 100 |
|  |  |  |  |  |  |
| **TIM2q** | 23.0 | 19.0 | 15.6 | 42.3 | 100 |
|  |  |  |  |  |  |
| **TIM3q** | 25.7 | 19.2 | 15.4 | 39.7 | 100 |
|  |  |  |  |  |  |
|  |  |  |  |  |  |
| **DeNovoTIM6** | 28.5 | 26.3 | 11.0 | 34.2 | 100 |
| **TIM2q-in** | 22.1 | 19.6 | 15.4 | 42.9 | 100 |
|  |  |  |  |  |  |
| **TIM2q-out** | 24.9 | 17.0 | 15.7 | 42.4 | 100 |
|  |  |  |  |  |  |
